# Relationship between Neural Activity and Executive Function: An NIRS Study

**DOI:** 10.1155/2014/734952

**Published:** 2014-03-09

**Authors:** Akira Yasumura, Masumi Inagaki, Kazuo Hiraki

**Affiliations:** ^1^Department of Developmental Disorders, National Institute of Mental Health, National Center of Neurology and Psychiatry, 4-1-1 Ogawahigashi, Kodaira, Tokyo 187-8553, Japan; ^2^Graduate School of Arts and Sciences, The University of Tokyo, Tokyo, Japan

## Abstract

*Objective*. This study examined the relationship between neural activity and executive function (EF) by near-infrared spectroscopy (NIRS). In addition, an oral reading span test (RST) was used to explore this association. *Methods*. Fifteen adults participated in the study. We used the RST and simple reading as the two tasks. *Results*. The RST score and cortical hemodynamic response in the left inferior frontal gyrus were significantly correlated. *Conclusion*. Based on the oral RST performance assessment and NIRS examination, the present findings suggest a relationship between EF and cortical activation.

## 1. Introduction

As an executive function (EF), working memory refers to the brain system that stores and manipulates information over brief periods and represents a key process for cognitive functions such as planning, reasoning, and problem solving [1]. The reading span test (RST) was developed and implemented to behaviorally measure individual differences in verbal working memory capacity employed by processing and storage functions during reading [2]. The RST is a dual-task paradigm in which participants are required to read a sentence and simultaneously remember target words. This span correlates with three reading comprehension measures, including the verbal scholastic aptitude test (SAT) and tests involving fact retrieval and pronominal references. These results contrast with those of traditional digit span and word span measures that do not correlate with comprehension [2]. The reader must also store the text theme, representation of the situation to which it refers, major propositions from preceding sentences, and a running, multilevel representation of the sentence that is currently being read. Thus, language comprehension is an excellent example of a task that demands extensive storage of partial and final products during complex information processing [2]. Recent neuroimaging studies have attempted to explore the neural basis of working memory systems based on Baddeley's theory [3]. It has been proposed that two types of working memory processes, which are executive control processes, are observed in distinct cortical structures located in the prefrontal cortex (PFC) and modality-specific buffers located in more posterior regions [4–6]. Activation of the dorsolateral PFC is observed when two types of tasks are performed together [7], such as during a task performed with a self-monitoring system [8] or during a task requiring executive control [9]. In this view, resource allocation during the span task would be controlled by the central executive system and exhibit increased activation in PFC and the anterior cingulate cortex while subjects performed the listening span test (LST), as shown by Osaka et al. [10]. Such activation represents the attention control system of the central executive system. Osaka et al. [11] referred to the neural substrate of central EF during the RST. However, because they used functional magnetic resonance imaging (fMRI) to measure neural activity, they performed silent RSTs. As a result, the correlation between the silent RSTs and reading comprehension is lower than that between oral RSTs and reading comprehension [2] because silent RSTs permit rehearsals and other internal processes. Oral reading suppresses internal processes such as rehearsal. Thus, the neural mechanisms of working memory must be explored using oral RST. The present study examined activity in the left and right frontal cortex during oral RST using near-infrared spectroscopy (NIRS). NIRS is a relatively new imaging technique for investigating cortical hemodynamic responses. Because oxygenated hemoglobin (oxy-Hb) and deoxygenated hemoglobin have different absorption spectra in the infrared range, changes in their concentrations can be calculated by projecting infrared light of two different wavelengths on the skull. The oxy-Hb concentration is generally coupled with regional cortical activation [12]. NIRS is noninvasive and robust against body movements, and it has been validated as a technique suitable for investigating neural mechanisms during oral reading.

## 2. Methods

### 2.1. Subjects

The subjects were 18 right-handed Japanese adults (14 men and 4 women; mean age ± standard deviation, 28.22 ± 5.50 years; range, 22–46 years). All subjects were in good health without any significant clinical history of physical or mental illness, and no subject was receiving any concomitant medication likely to interfere with the results.

Informed consent was obtained from all subjects in accordance with the protocol approved by the Tokyo University Review Board.

### 2.2. Tasks

We used an RST and simple reading as the two tasks. Seventy unrelated sentences were used for the RST, as described previously [13]. The sentences were taken from Japanese high-school textbooks because the difficulty level was supposed to be appropriate for our adult subjects. Each sentence was presented in a written format and comprised of 20–30 Japanese characters (including Kanji, Hiragana, and Katakana).

We used four-sentence conditions: two sentences, three sentences, four sentences, and five sentences. As an example of the two-sentence conditions, the subject had to read the first and second sentences aloud (in that order) when they were presented. Then, the subject needed to recall two target words included in each sentence. Thus, the subject needed to remember five target words in the five-sentence condition.

English versions of the RST often designate the last word of each sentence as the target word [2], but we avoided this common practice because most Japanese sentences have verbs in the final position, which might be easily predictable. Therefore, we set the target words at various positions. However, we tended to equal the number of word parts and underlined them using a red marker. Each sentence for the reading task was the same as that for the RST, but no words were underlined.

### 2.3. Procedure

The two tasks were presented on a computer screen. Each subject took a seat in front of a table on which a laptop computer with a 15-inch liquid crystal screen was placed. The distance between the subject's eyes and the screen was approximately 60 cm. The order of each task was counterbalanced across subjects.

**Figure 1:**
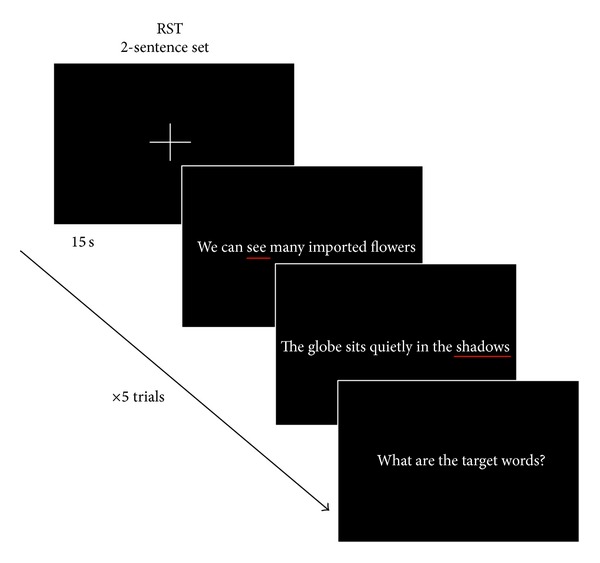
Time course of the RST conditions for the two-sentence set. The rest period was 15 s. Five trials were conducted.

Figure 1 shows the time course of the RST conditions. First, the subject was required to gaze at a cross in the center of the screen for 15 s; this was considered the rest period. Second, the subject read a set of sentences aloud and recalled the target word from each sentence at the end of the set. The subjects were allowed to read the sentences at their own pace. However, the recall time was 5 s per word. Therefore, the time limits were 10 s for two sentences and 25 s for five sentences. Five trials were conducted.

### 2.4. Near-Infrared Spectroscopy Recording

**Figure 2:**
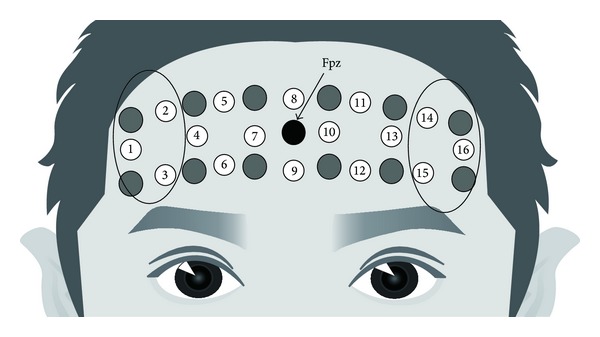
Cortical responses were obtained from 16 locations. The center of the probe matrix was placed on Fpz (midpoint between Fp1 and Fp2) in accordance with the international 10/20 system used in electroencephalography.

Changes in oxy-Hb concentrations were recorded using an OEG-16 apparatus (Spectratech Inc., Tokyo, Japan) with 16 channels. Two wavelengths of approximately 770 and 840 nm were used to estimate changes in oxy-Hb concentrations. Six emission and six detector probes were arranged in a 6 (wide) × 2 (long) matrix on the subject's forehead. Thus, cortical responses were obtained from 16 locations. The center of the probe matrix was placed on Fpz (midpoint between Fp1 and Fp2) in accordance with the international 10/20 system used in electroencephalography. The probe in the bottom left corner was placed around F7, and the right probe was placed around F8 (Figure 2).

The emission probes were located 3 cm apart from the detector probes. In this manner, we could measure changes in oxy-Hb concentrations at a depth of approximately 3 cm below the scalp. The temporal resolution was set every 650 ms.

### 2.5. Statistical Analysis

A bandpass filter was set for the NIRS data at 0.033–5 Hz in the two-sentence condition, 0.022–5 Hz in the three-sentence condition, 0.017–5 Hz in the four-sentence condition, and 0.013–5 Hz in the five-sentence condition using fast Fourier transform to reject artifacts caused by minor subject movements. To determine oxy-Hb concentrations, changes in the self-face specific region of the lateral PFC and the region of interest (ROI) for the NIRS data were arranged into two regions: (1) right inferior frontal gyrus (R-IFG: channels 1, 2, and 3) and (2) the left inferior frontal gyrus (L-IFG: channels 13, 14, and 15) (Figure 2). Changes in oxy-Hb concentrations in each ROI were averaged individually [14]. To increase the signal-to-noise ratio, data from each channel were converted into a *z*-score because raw data cannot be compared directly across subjects and channels [15]. The *z*-score was calculated using the mean and standard deviation of changes in the oxy-Hb concentrations during the last 10 s of the rest period. Consequently, the mean and standard deviation were adjusted to a *z*-score of 0 and 1 for every channel, respectively. Subjects with a score of more than 2 standard deviations were excluded because of the possibility of motion artifacts. As a result, 15 subjects were included for the final analysis.

RST scores were calculated using a previously described method [13]. The scoring method was adapted from a previous study [2]. If a subject answered more than three target words correctly, then this represented a passing condition and 3–5 points were allotted. If the subject answered two target words correctly, then 0.5 points were allotted. Therefore, if three correct answers were given in the three-sentence condition and two correct answers were given in the four-sentence condition, then the score was 3.5 points.

Correlation between task performance (RST score) and *z*-score of the NIRS response was analyzed. We used the average *z*-score for each condition to examine changes in oxy-Hb concentrations that were related to the number of sentences.

## 3. Results

The mean score for recalling the target words was 2.8 (SD = 0.5) for the 15 subjects (3 women).

**Figure 3:**
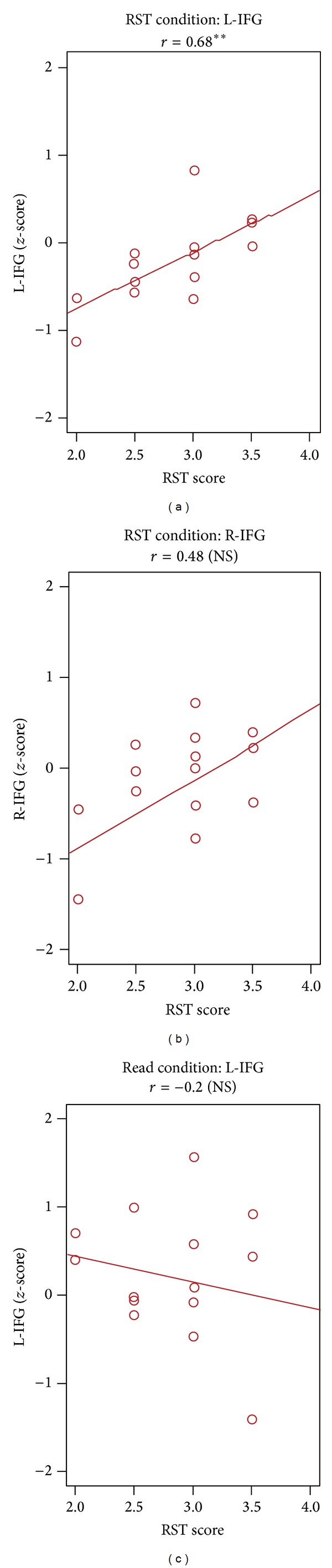
Under the RST conditions, the correlation between the *z*-score for L-IFG and RST score was significant (*r* = 0.68, *P* < 0.01). Under the reading condition, the correlation between the *z*-score for L-IFG and RST score was not significant (*r* = −0.2, *P* = 0.5). Moreover, we examined the differences between brain regions during the same RST conditions. The correlation between the *z*-score and RST score was not significant in R-IFG (*r* = 0.48, *P* = 0.07).

One-way analysis of the variance was performed using the *z*-score to compare the effect of sentence condition on the RST. As a result, we revealed no main effects and no interaction for R-IFG (*F* = 1.5, *P* = 0.2) or L-IFG (*F* = 1.2, *P* = 0.3). Therefore, we averaged the *z*-scores for all conditions as brain activity. The relationships between brain activity and task performance were analyzed (Figure 3). First, we examined the differences between the R-IFG and L-IFG regions during the RST. The correlations between brain activity and the RST scores were not significant in R-IFG (*r* = 0.48, *P* = 0.07) but were significant in L-IFG (*r* = 0.68, *P* < 0.01). Second, we examined the differences between the tasks. The correlation between brain activity in L-IFG and the RST scores was not significant for the reading task (*r* = −0.2, *P* = 0.5).

## 4. Discussion

Our results revealed a correlation between brain activity in L-IFG and task performance on oral RST performance, suggesting that EF involves brain activity in L-IFG. Silent RST and LST were performed in a previous fMRI study. By using oral reading tasks in the present study, we suppressed internal processes such as rehearsal.

Many studies have divided subjects into two groups based on their working memory scores: high-span subjects and low-span subjects [10, 11, 13]. However, EF may be based on a normal distribution. Then individual differences in EF can be illustrated using a slope. In the present study, we presented this slope as a correlation between brain activity in L-IFG and task performance.

The present study may also contribute to our understanding of developmental disorders such as attention deficit hyperactivity disorder (ADHD). Children with ADHD experience difficulty with EF tasks, and some patients with ADHD display weaker prefrontal activation on the stop-signal task [16]. In addition, recent neuroanatomical research suggests that children with ADHD exhibit a marked delay in the maturation of prefrontal areas [17]. These studies suggest that ADHD patients may have functional and anatomical deficits in PFC. However, there have been few brain imaging studies of young children with ADHD. Thus, our functional developmental approach, along with the anatomical evidence, may contribute to determining when and how children with ADHD exhibit earlier neural symptoms.

The attention control mechanism is involved in inhibiting information, and central EF is mainly responsible for this role. Because Baddeley suggested that PFC is related to the executive control function system, many studies have investigated the relationship between PFC and central EF [3]. The present NIRS study clearly established a relationship between L-IFG and a working memory task; therefore, we have provided strong evidence for a neural basis of central EF.

The NIRS technique has some shortcomings compared to other neuroimaging techniques such as fMRI. First, NIRS detects hemodynamic changes only at the brain surface (approximately 2 cm beneath the skull). Thus, subcortical responses cannot be examined by NIRS. Second, NIRS offers relatively low spatial resolution compared with fMRI; therefore, precise analyses are limited. Despite these shortcomings, NIRS is increasingly becoming a key modality in developmental neuroscience involving studies of newborn infants [18], preschool children [19], and school-aged children [15] because of its excellent safety and robustness against body movements. Our previous study revealed the deficit of EF in children with autism spectrum disorder [20]. We believe that we can predict the power of EF by NIRS in children who do not know all language characters.

## 5. Conclusions

We examined the neural basis of EF by NIRS and a RST as a working memory task. Behavioral performance and neural activity in L-IFG during oral RST were significantly correlated. This is the first study to demonstrate the relationship between oral RST performance and cortical activation by NIRS.
